# Evaluation of Cisplatin-Induced Acute Renal Failure Amelioration Using Fondaparinux and Alteplase

**DOI:** 10.3390/ph16070910

**Published:** 2023-06-21

**Authors:** Mohamed S. Abdel-Bakky, Anas S. A. Aldakhili, Hussein M. Ali, Ali Y. Babiker, Ahmad H. Alhowail, Salman A. A. Mohammed

**Affiliations:** 1 Department of Pharmacology and Toxicology, College of Pharmacy, Qassim University, Buraydah 51452, Saudi Arabia; m.abdelbakky@qu.edu.sa (M.S.A.-B.); 431114053@qu.edu.sa (A.S.A.A.); hu.ali@qu.edu.sa (H.M.A.); aalhowail@qu.edu.sa (A.H.A.); 2 Department of Pharmacology and Toxicology, Faculty of Pharmacy, Al-Azhar University, Cairo 11884, Egypt; 3 Department of Biochemistry, Faculty of Medicine, Al-Azhar University, Assiut 71524, Egypt; 4 Department of Medical Laboratories, College of Applied Sciences, Qassim University, Buraydah 51452, Saudi Arabia; alibabkr99@gmail.com

**Keywords:** fondaparinux, cisplatin, alteplase, acute kidney failure, apoptosis, protein expression, in vivo study, anticoagulant agent, platelet count, A Disintegrin and Metalloproteinases-10, protease-activated receptor-2

## Abstract

Acute renal failure (ARF) is a deleterious condition with increased mortality or healthcare costs or dialysis-dependent end-stage renal disease. The study aims to compare prophylaxis with fondaparinux (Fund) vs. treatment with alteplase (Alt) in ameliorating cisplatin (Cis)-induced ARF. Sixty male mice were equally divided randomly into six groups of control, Cis, Alt, and Cis + Alt groups receiving normal saline for 10 days. All four groups except for the control received Cis (30 mg/kg, i.p.) on day 7, and 6 h later, both the Alt groups received Alt (0.9 mg/kg, i.v.). The animal groups Fund and Fund + Cis received Fund (5 mg/kg, i.p.) for 10 days, and the Fund + Cis group on day 7 received Cis. All the animal groups were euthanized 72 h after the Cis dose. The Fund + Cis group showed significantly increased expression levels of platelet count, retinoid X receptor alpha (RXR-α) and phosphorylated Akt (p-Akt) in addition to decreased levels of urea, blood urea nitrogen (BUN), uric acid, white blood cells (WBCs), red blood cells (RBCs), relative kidney body weight, kidney injury score, glucose, prothrombin (PT), A Disintegrin And Metalloproteinases-10 (ADAM10), extracellular matrix deposition, protease-activated receptor 2 (PAR-2), and fibrinogen expression when compared to the Cis-only group. Meanwhile, the Cis + Alt group showed increased caspase-3 expression in addition to decreased levels of urea, BUN, uric acid, WBCs, RBCs, glucose, platelet count and PT expression with a marked decrease in PAR-2 protein expression compared to the Cis group. The creatinine levels for both the Fund + Cis and Cis + Alt groups were found to be comparable to those of the Cis-only group. The results demonstrate that the coagulation system’s activation through the stimulation of PAR-2 and fibrinogen due to Cis-induced ADAM10 protein expression mediated the apoptotic pathway, as indicated by caspase-3 expression through the p-Akt pathway. This is normally accompanied by the loss of RXR-α distal and proximal tubules as lipid droplets. When the animals were pre-treated with the anticoagulant, Fund, the previous deleterious effect was halted while the fibrinolytic agent, Alt, most of the time failed to treat Cis-induced toxicity.

## 1. Introduction

Acute renal failure (ARF) is a deleterious condition with high mortality rates, substantial healthcare costs, and a higher amount of dialysis-dependent patients. ARF is diagnosed by an increase in serum creatinine (Sr. Cr.) and reduced urine production [1]. The Kidney Disease: Improving Global Outcomes (KDIGO) guidelines define ARF as elevated Sr. Cr. within 2 days, an escalation of Sr. Cr. by 1.5 × 1 week, or reduced urine (<0.5 mL/kg/day) for at least six hours [2]. ARF can cause the accumulation of water, sodium, and other metabolic products as well as electrolyte imbalances with clinical and cellular phases, including initiation, extension, maintenance, and recovery.

The early initiation phase of ARF is characterized by injury to the epithelial cells found in the renal tubules, particularly the thick ascending medullary limb of the Henlé loop, and the straight proximal tubules (PTs). The severity and duration of the ischemic injury determine the extent of these changes, and the activation of epithelial and possibly endothelial cells initiates the inflammatory cascade [3]. Patients with ARF may also experience dysfunctional hemostasis, such as an increased risk of bleeding and venous and arterial thromboembolism [4].

The activation of epithelial and possibly endothelial cells during the early initiation phase of ARF results in the upregulation of a variety of chemokines and cytokines that are instrumental in initiating the inflammatory cascade [5]. Many nephrotoxic agents can induce ARF including angiotensin-converting enzyme (ACE) inhibitors, angiotensin receptor blockers (ARBs), certain antibiotics, cisplatin (Cis), contrast dye, and recreational drugs such as cocaine [6]. An essential antineoplastic agent such as Cis is mainly effective for the treatment of ovarian and testicular cancers [7]. The accumulation of Cis in the kidney has been shown to a greater degree than in other organs [8]. Although Cis has a suppressing effect on tumors, its use is limited due to its nephrotoxic, ototoxic, neurotoxic and hepatotoxic effects. Cis is bio-transformed into highly reactive thiols that injure tubule cells, destroy the cell membrane and induce tubular dysfunction through mechanisms such as the production of reactive oxygen species (ROS), oxidative and nitrative stress, hydroxyl radicals, lipid peroxidation, DNA fragmentation, inflammation, and hypoxia, ultimately resulting in the activation of apoptotic or necrotic pathways [9].

A Disintegrin And Metalloproteinases-10 (ADAM10) is a protein in the ADAMs family that functions as a sheddase, cleaving transmembrane proteins’ extracellular regions. The A Disintegrin And Metalloprotease (ADAM) family includes 22 members, but only 12 encode active enzymes, with ADAM10 being one of the most characterized members [10]. A constitutive expression of ADAM10 is found as a transmembrane in different cells, such as immune cells and renal tubule cells, with at least 40 substrates [11]. The activation of ADAM10 initiates an inflammatory and fibrotic response to renal diseases, including ARF, and recent studies have shown a link between ADAM10 and coagulation in kidney disease [12,13,14,15,16].

Retinoid X receptor alpha (RXR-α) is a type of nuclear receptor that binds to retinoids, which are compounds similar to vitamin A [17]. The binding of RXR-α and retinoic acid receptor (RAR) activates nuclear pathways that regulate gene expression, controlling important cellular functions such as apoptosis, proliferation, or differentiation [18]. An important role of RXR-α has been observed in renal diseases and is mainly expressed in liver hepatocytes, hepatic stellate cells, and renal proximal and distal tubules (DT). The RAR family is interested in regulating ADAM10 for its therapeutic potential [19]. The RAR-α and β can activate the ADAM10 promoter. The RXR-α plays a vital role in various renal diseases, and cisplatin (Cis) low doses increased its expression in the head and neck squamous cell carcinoma (HNSCC) cell line and rat model [20]. However, Cis treatment deactivated peroxisome proliferator-activated receptor (PPAR-α) by reducing PPAR-α/RXR-α binding activity in mice [21].

Protease-activated receptor 2 (PAR-2) is a type of membrane receptor that can be released by proteases, particularly in the digestive system [22]. Its activation stimulates the release of inflammatory cytokines and chemokines from various cells, leading to pro-inflammatory effects. PAR-2 may also control vascular reactivity and regulate blood pressure and electrolyte balance in the kidney collecting ducts [23,24].

Proteins such as p-AKT, which are involved in the PI3K pathway control important cellular roles such as cell proliferation, differentiation, and apoptosis. Tyrosine kinases and G-protein coupled receptors initiate the activities of p-AKT and are crucial objects in cancer treatment due to their ability to promote cell survival and resistance to chemotherapy [25,26]. The role of ADAM10 is demonstrated in the progression of various renal diseases, including acute kidney injury, indicating it is an excellent therapeutic target for many kidney disorders. There is a scarcity of publications on the expression and function of ADAM10, RXR-α, PAR-2, and p-Akt pathways in the ARF and their functional role in developing inflammatory kidney diseases. Coagulation system activation is one of the new strategies, and its role in the pathophysiology of ARF was investigated in a few studies. The correlation between the ADAM10 and the coagulation system is not demonstrated in general or in CP-induced renal toxicity in particular.

Fondaparinux (Fund), a synthetic selective inhibitor of factor Xa, plays its role in controlling pulmonary embolism (PE) and deep vein thrombosis (DVT) and is often prescribed for post-operative patients of major abdominal, hip, or knee surgeries [27]. One of the limitations of heparin is causing thrombocytopenia which does not occur in the case of Fund [28], and it has 100% bioavailability when administered subcutaneously with a relatively long half-life of 17–21 h. The fund is excreted through the kidneys and has fewer side effects compared to other oral anticoagulants [29]. However, to our knowledge, there is no report on the possible protective role of the Fund in Cis-induced ARF.

On the other hand, Alteplase (Alt) is a recombinant DNA-manufactured thrombolytic agent that is used for acute myocardial infarction, acute ischemic stroke, occluded catheters, PE, DVT and peripheral arterial occlusive disease [30]. Alteplase converts plasminogen into plasmin, which lyses both fibrinogen and fibrin. It is administered intravenously and metabolized by the liver, with a half-life of 5 to 72 min [31]. A recent study has confirmed the efficacy and safety of Alt in the treatment of moderate/severe COVID-19-induced acute respiratory distress syndrome. In addition, it has been found to decrease pulmonary fibrosis in rat models induced by bleomycin and increase levels of antioxidants while reducing hydroxyproline [32].

In summary, both Fund and Alt are effective anticoagulants with different mechanisms of action and administration routes. While Fund has fewer side effects and is excreted through the kidneys, Alt is a thrombolytic agent that can be used to treat multiple conditions. Furthermore, it was reported that the safety and efficacy of reteplase or Alt mixed with aspirin for hyper-acute cerebral infarction treatment were better than monotherapy of reteplase or Alt [33].

No data exist about the possible ameliorating effect of fund or Alt against Cis-induced ARF. Therefore, the current study aims to clarify the possible ameliorating role of Fund or Alt against Cis-induced ARF. The study aims to compare prophylaxis with Fund vs. treatment with Alt in ameliorating Cis-induced ARF in addition to establishing the relationship between ADAM10, RXR-α, and PAR-2 and the role of AKT.

## 2. Results

### 2.1. Effect of Fondaparinux (Fund) or Alteplase (Alt) with/without Cisplatin on the Kidney

**Figure 1:**
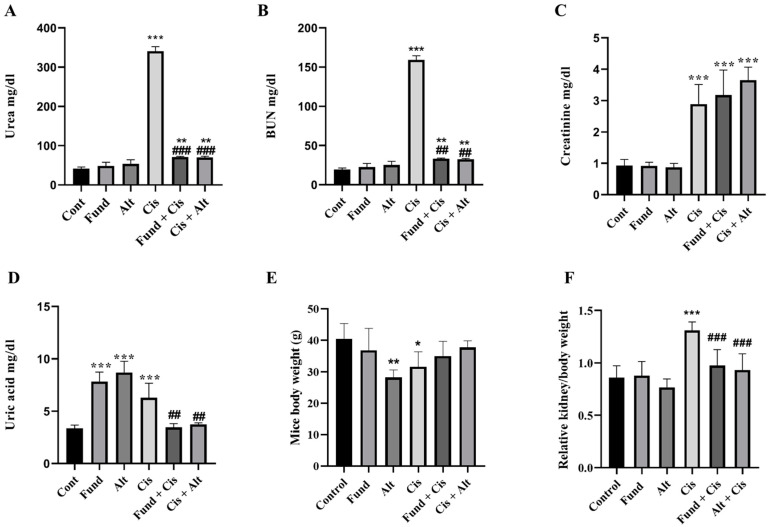
Effect of cisplatin (Cis) and/or fondaparinux or alteplase on kidney function; urea (**A**), BUN (**B**), creatinine (**C**), uric acid (**D**), mice body weight (**E**) and relative kidney/body weight (**F**). Each bar represents the mean of 10 mice ± S.E.M. Statistical analysis was performed using one-way ANOVA followed by Tukey–Kramer multiple comparisons test where: * *p* < 0.05, ** *p* < 0.01 and *** *p* < 0.001 are significantly different when equated with the control group, ^##^ *p* < 0.01 and ^###^ *p* < 0.001 are significantly different compared to the Cis group.

Cisplatin-induced nephrotoxicity (Cis) mice demonstrated increased urea (*p* < 0.001) and blood urea nitrogen (BUN) (*p* < 0.001) levels by 723.58% and 721.46%, respectively, compared to the control (Figure 1A,B). The Fund (Not significant, *N.S.*), Alt-treated (*N.S.*), Cis-pre-treated with Fund (Fund + Cis) (*p* < 0.01) or Cis + Alt (*p* < 0.01) animals showed a change in urea (16.94%, 30.2%, 71.65%, and 68.82%, respectively) and BUN (16.66%, 29.87%, 71.25% and 67.18%, respectively) compared to the control group. In addition, Cis-pre-treated with Fund (Fund + Cis) (*p* < 0.001) or Cis + Alt (*p* < 0.001) groups showed decreased urea by 79.1% and 79.68% and BUN by 78.46% and 55.17%, respectively, compared to Cis mice.

Similarly, creatinine was found to be significantly increased in the Cis (*p* < 0.001), Fund + Cis (*p* < 0.001) and Cis + Alt (*p* < 0.001) groups by 214.13%, 244.56% and 296.73%, respectively, and comparable in the Fund (−1.08%, *N.S.*) and Alt-treated (−5.43%, *N.S.*) groups compared to the control. In addition, the Fund + Cis (*N.S.)* and Cis + Alt (*N.S.)* group’s creatinine levels increased by 9.68% and 26.29%, respectively, when equated to Cis mice (Figure 1C).

The uric acid levels of the Cis (*p* < 0.001), Fund (*p* < 0.001), Alt (*p* < 0.001), Fund + Cis (*N.S.*) and Cis + Alt (*N.S.*) groups increased by 88.05%, 133.73%, 159.70%, 2.98% and 10.44%, respectively, when compared to the control group, whereas the Fund + Cis (*p* < 0.01) and Cis + Alt (*p* < 0.01) groups showed reduced uric acid levels by 45.23% and 41.26%, respectively, compared to the Cis group (Figure 1D).

The body weights for the Cis (*p* < 0.05), Fund (*N.S.),* Alt (*p* < 0.01), Fund + Cis (*N.S.*) and Cis + Alt (*N.S.*) groups decreased by −21.71%, −9.00%, 30.24%, −13.46% and −6.84% compared to the control group. In addition, the administration of Fund or Alt with Cis showed insignificantly increased BW by 10.62% and 19.11%, respectively, as compared to Cis-induced nephrotoxicity (Figure 1E). The relative kidney/body weight ratio of the Cis group (*p* < 0.001) was found to be increased by 51.27% and comparable for the Fund (1.5%, *N.S.*), Alt (−11.54%, *N.S.*), Fund + Cis (12.00%, *N.S.*) and Cis + Alt (7.73%, *N.S.*) groups when equated to the control. In addition, the Fund + Cis (*p* < 0.001) and Cis + Alt (*p* < 0.001) groups showed reduced weight ratios by 25.95% and 28.77%, respectively, compared to the Cis group (Figure 1F). Notably, even though food and water consumption over the experimental procedure remained incomplete, the consumption of water and food, in general, was observed to be lower in the Alt, Cis and Cis +Alt groups.

### 2.2. Effect of Cisplatin Time Course on White Blood Cells (WBCs), Red Blood Cells (RBCs), Platelets (PLT), Prothrombin Time and Glucose Level

The results presented in Table 1 showed that the normal WBCs count for the Cis (*p* < 0.01), Fund (*N.S.*), Alt (*N.S.*), Fund + Cis (*p* < 0.001) and Cis + Alt (*p* < 0.001) groups decreased by −10.16%, −8.37%, −24.24%, −47.05% and −69.34% compared to the control group. Similarly, the WBCs count decreased by −30.11% and −59.52% for both the Fund + Cis (*p* < 0.01) and Cis + Alt (*p* < 0.001) groups, respectively, compared to the Cis group.

A significant elevation in RBC count was observed for Cis (*p* < 0.001) mice by 15.01%, while the Fund (*N.S.*), Alt (*N.S.*), Fund + Cis (*N.S.*), and Cis + Alt (*N.S.*) groups demonstrated an insignificant change of −2.09%, 7.87%, −0.54% and 0.73%, respectively, compared to the control group. On the other hand, the RBC count decreased by −13.53% and −12.42% for the Fund + Cis (*p* < 0.001) and Cis + Alt (*p* < 0.001) groups, respectively, compared to Cis mice.

The platelet count (PLT) in the Cis (*p* < 0.001), Fund (*p* < 0.05), Alt (*p* < 0.001), Fund + Cis (*p* < 0.01) and Cis + Alt (*p* < 0.001) groups showed a significant reduction by −41.72%, −7.29%, −63.77%, −8.17% and −57.94%, respectively, compared to the control group. Furthermore, the PLT count in the Fund + Cis (*p* < 0.001) and Cis + Alt (*p* < 0.001) groups increased and decreased significantly by 57.56% and −27.84%, respectively, compared to Cis mice.

The prothrombin time (PT) for the Cis (*p* < 0.001), Fund (*p* < 0.05), Alt (*p* < 0.05), Fund + Cis (*p* < 0.01) and Cis + Alt (*p* < 0.001) groups was found to be increased by 74.18%, 27.44% 23.25%, 34.18% and 32.32%, respectively, when equated to the control. On the contrary, the PT levels of the Fund + Cis (*p* < 0.01) and Cis + Alt (*p* < 0.01) groups decreased by 22.96% and 24.03%, respectively, when equated to the Cis group.

The level of glucose in the Cis (*p* < 0.05) group showed an elevation of 24.26%, while the Fund, Alt, Fund + Cis (*p* < 0.01) and Cis + Alt (*p* < 0.001) groups levels were comparable by a change of 2.34% and −1.30% when equated to the control group. Both the Fund + Cis (*p* < 0.001) and Cis + Alt (*p* < 0.001) groups showed decreases in glucose levels by 46.11% and 56.38%, respectively, compared to the Cis group.

### 2.3. Effect of Cis with or without Fund or Alt on ADAM10 Protein Expression in Renal Mice Tissues

**Figure 2:**
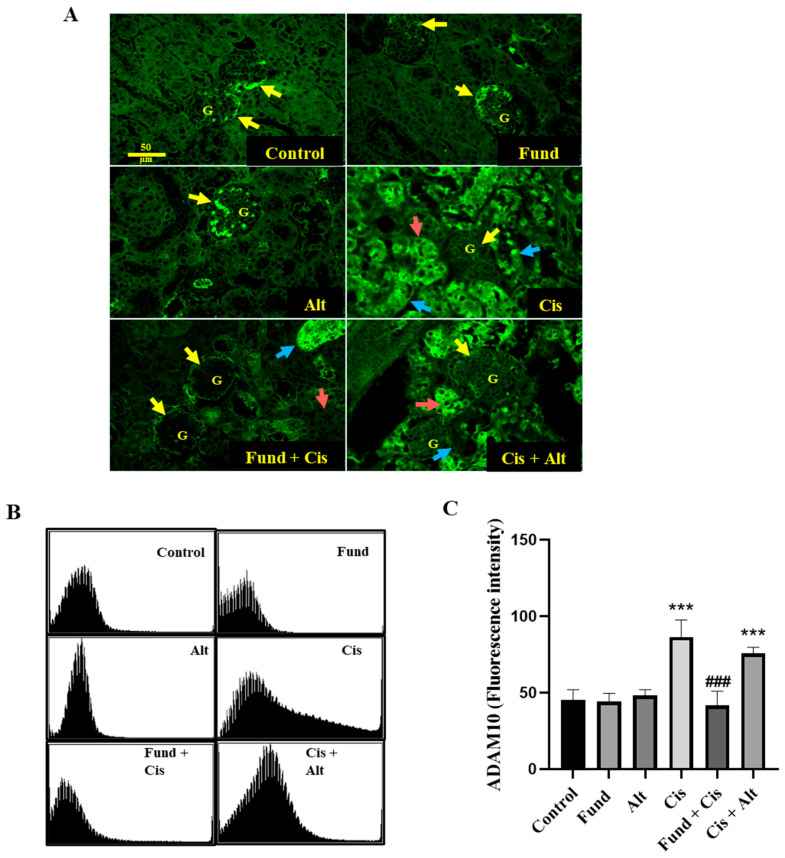
Effect of cisplatin with or without fondaparinux or alteplase on ADAM10 protein expression in kidneys. Yellow, red and blue arrows refer to glomeruli (G), cortical distal tubule (DT), and proximal tubule (PT), respectively (**A**), and histogram presentation (**B**) and fluorescence intensity graphical presentation (**C**). Data are expressed as mean ± SEM, where: *** *p* < 0.001 significantly different from the control group, ^###^ *p* < 0.001 significantly when compared to cisplatin mice, using one-way ANOVA followed by the Tukey–Kramer multiple comparisons test value. Yellow arrows and arrowheads refer to DT and PT, respectively. Scale bar = 50 μm.

In Figure 2, the immunofluorescence staining of kidney tissues revealed basal constitutive ADAM10 protein expression in the glomerular podocytes (yellow arrow) with a negative expression of ADAM10 in the cortical tubules including the PT, DT, collecting duct, and Henle loop of the control, Fund, and Alt -treated groups. The Cis group showed a marked reduction in the glomerular ADAM10 expression with an increased expression in the cortical DT (red arrow) and PT (blue arrow) tubules. In addition, the Fund + Cis and Cis + Alt groups showed a strong reduction in ADAM10 expression in the glomeruli (G, yellow arrow) and DT (red arrow) with an increased expression in the PT (blue arrow) as well (Figure 2A,B). The level of ADAM10 in the Cis (*p* < 0.001) and Cis + Alt (*p* < 0.001) groups showed an elevation of 89.64% and 66.43%, while the Fund (*N.S.*), Alt (*N.S.*) and Fund + Cis (*N.S.*) groups levels were comparable by a change of −3.13%, 5.86%, and −8.58% when equated to the control group (Figure 2C). The expression of ADAM10 decreased significantly in the Fund + Cis (*p* < 0.001) group, while it remained insignificant in the Cis + Alt (*N.S.*) groups by −51.79% and −12.23%, respectively, compared to the Cis group.

### 2.4. Effect of Cis with/without Fund or Alt on RXR-α Protein Expression in Renal Mice Tissues

**Figure 3:**
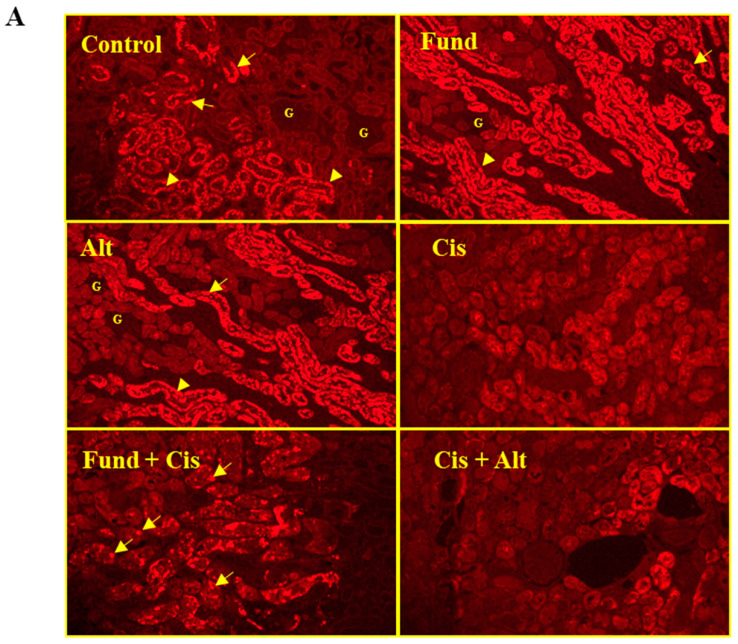

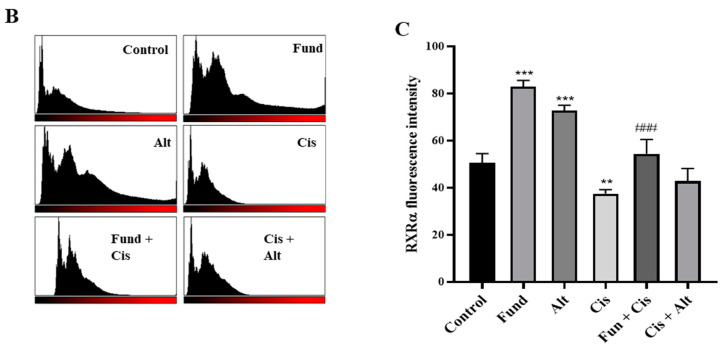
Effect of Cis with or without Fund or Alt on RXR-α protein expression in mice renal tissues using immunofluorescence method (**A**) and histogram presentation (**B**) and fluorescence intensity graphical presentation (**C**). Data are expressed as mean ± SEM, where: ** *p* < 0.01 and *** *p* < 0.001 are significantly different from the control group, ^###^ *p* < 0.001 significantly when equated to cisplatin mice, using one-way ANOVA followed by the Tukey–Kramer multiple comparisons test value. Yellow arrows and arrowheads refer to DT and PT, respectively. Scale bar = 100 μm.

In control kidneys, a constitutive RXR-α expression was seen in the principal cells of DT (yellow arrows) and PT (yellow arrowhead) tubules with no RXR-α protein expression in the G using immunofluorescence staining. The Cis group showed a marked reduction in RXR-α with translocation from the basolateral into the apical site of DT and PT. Partial restoration of RXR-α protein was observed in DT and PT, while on the contrary, its decline as lipid droplets in other tubular sites (yellow arrows) was demonstrated significantly by the Fund + Cis group and insignificantly by the Cis + Alt group (Figure 3A).

The histogram (Figure 3B) and fluorescence intensity (Figure 3C) further confirm the Cis effect with/without Fund or Alt. The RXR-α expression in the Cis (*p* < 0.01) and Cis + Alt (*N.S*) groups decreased by −25.80% and −15.44% and increased for the Fund (*p* < 0.001), Alt (*p* < 0.001), and Fund + Cis (*N.S*) groups by 63.76%, 43.89% and 7.63%, respectively, when equated to the control group. Similarly, the RXR-α expression in both the Fund + Cis (*p* < 0.001) and Cis + Alt (*N.S*) groups increased by 45.09% and 13.96%, respectively, compared to the Cis group.

### 2.5. Effect of Cis with/without Fund and Alt on the Structural Architecture

**Figure 4:**
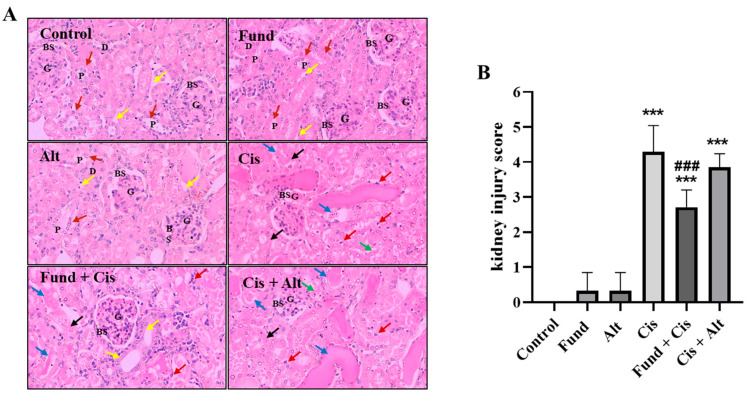

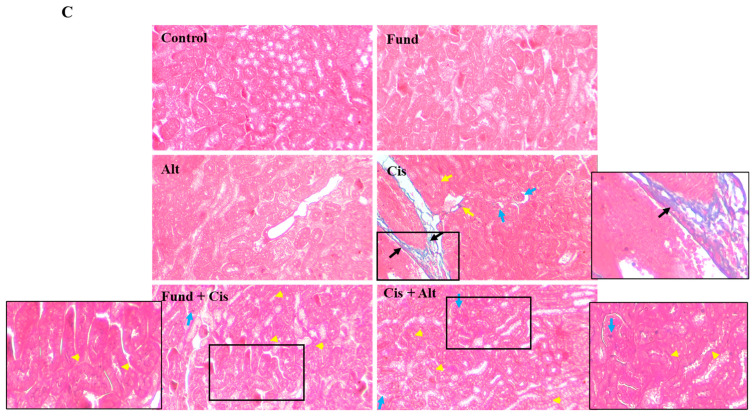
Photomicrograph staining using hematoxylin and eosin (**A**), kidney injury scores (**B**) and extracellular matrix deposition using the Mason trichrome stain (**C**) of mice renal tissues. (**A**): In the control, Fund, and Alt-treated groups, red and yellow arrows refer to brush borders (BB) and interstitium, while in the remaining groups, red, green, blue and black arrows refer to the epithelial lining (EL), partial loss of BB, intratubular hyaline casts (ITHC) and edematous EL, respectively. (**B**): Kidney injury score for control and groups treated with Cis in the presence or absence of Fund or Alt. (**C**): Arrows and arrowhead refer to the blue-colored extracellular matrix (ECM) deposition in the tubular interstitial spaces (yellow arrow) in the vicinity of the blood vessel (black arrow), apical part of the distal PT and DT (yellow arrowhead), and G (blue arrow). Magnification 400× and 200×, respectively, for A and C. Data in the graph representing means ± SEM (n = 6), where *** *p* < 0.001 is considered statistically different compared to the tissue sample of the control and ^###^ *p* < 0.001 is considered statistically different compared with the Cis group.

In Figure 4A, the control, Fund, and Alt-treated groups display kidney sections with a normal average G, normal PT with contact brush borders (BB, red arrow), normal Bowman’s spaces (BS), average DT, and average interstitium (yellow arrow).

The Cis group showed kidney tissues with glomerular atrophy (GA), wide BS, and PT demonstrating markedly apoptotic epithelial lining (EL, red arrows) with a partial loss of BB (green arrows), while others are dilated with intra-tubular hyaline casts (ITHC, blue arrows) and edematous EL (black arrows) (Figure 4A).

Moreover, the Fund + Cis group revealed kidney tissues with normal-sized G, average BS, and PT exhibiting a lower number of apoptotic cells (red arrows) with average EL (yellow arrow) and preserved BB (blue arrow) dilated with ITHC (blue arrows). In addition, the Cis + Alt group exhibited GA, wide BS, marked apoptotic EL in PT (red arrows) with a partial loss of BB (green arrows), some PT dilated with ITHC (blue arrows), and edematous EL (black arrows) (Figure 4A). Furthermore, the administration of Fund but not Alt in the presence of Cis significantly reduced renal injury scores compared to Cis-induced renal injury. The level of kidney injury score (KIS) in the Cis (*p* < 0.001), Fund (*N.S.*), Alt (*N.S.*), Fund + Cis (*p* < 0.01) and Cis + Alt (*p* < 0.001) groups showed a significant increase of 428%, 33%, 33%, 274% and 385%, respectively, compared to the control group (Figure 4B). Furthermore, the KIS in the Fund + Cis (*p* < 0.001) and Cis + Alt (*N.S.*) groups decreased by −36.07% and −10.17%, respectively, compared to Cis mice.

The Mason trichrome stain of renal tissues from the control, Fund, and Alt groups lacked any extracellular matrix (ECM) deposition in the renal tissues. The Cis- and Cis + Alt-treated mice showed ECM deposition (blue colored as it occurs normally at the beginning of fibrosis reaction) in the tubular interstitial spaces (yellow arrow) in the vicinity of the blood vessel (black arrow), apical part of the distal PT and DT (yellow arrowhead), and G (blue arrows). On the contrary, the Fund + Cis group showed less ECM deposition (Figure 4C).

### 2.6. Effect of Cis with/without Fund and Alt on p-AKT and Caspase-3 Proteins Expression in Mice Renal Tissues

**Figure 5:**
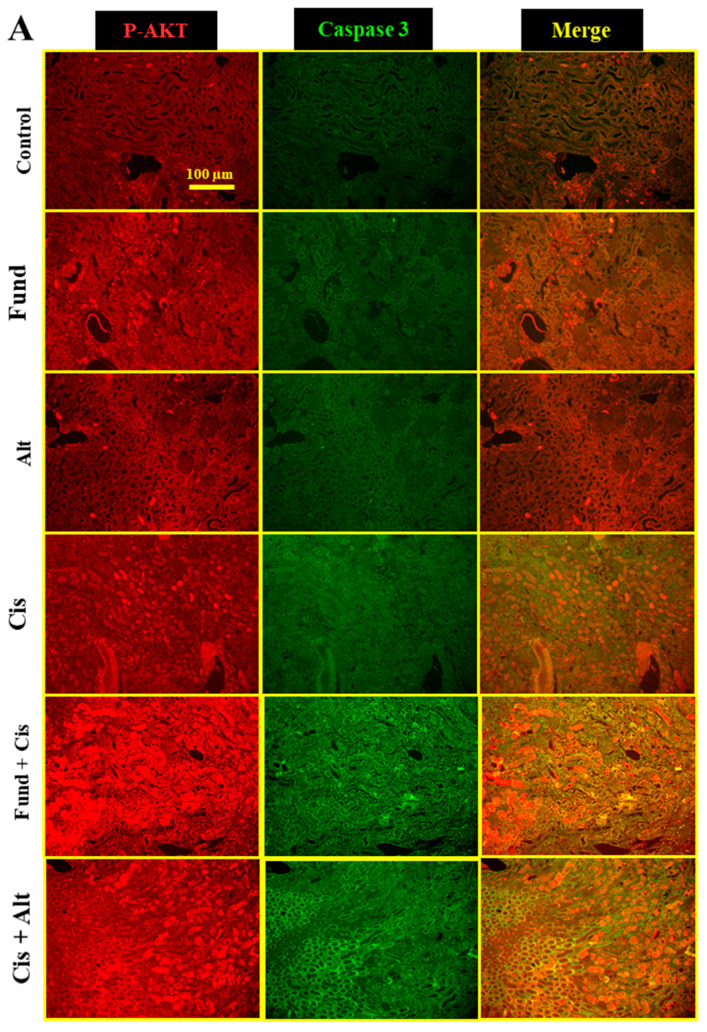

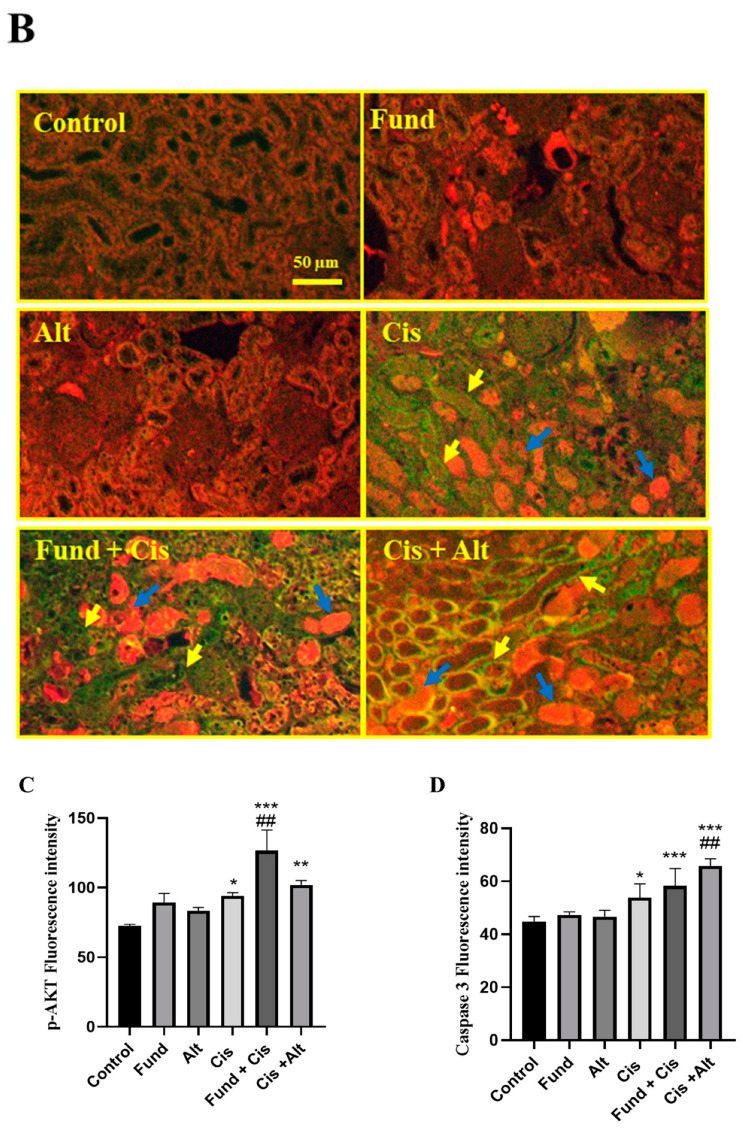
Effect of cis with/without Fund or Alt on p-AKT and caspase-3 proteins expression in mice renal tissues using double immunofluorescence staining, 200× (**A**) and 400× (**B**), fluorescence intensity graphical presentation for p-AKT (**C**) and caspase-3 (**D**) in mice groups were analyzed and blotted. Data are expressed as mean ± SEM, where: * *p* < 0.05, ** *p* < 0.01, and *** *p* < 0.001 significantly when equated to the control group, ^##^ *p* < 0.01 significantly different from cisplatin mice, using one-way ANOVA followed by the Tukey–Kramer multiple comparisons test value. Blue and yellow arrows refer to the expression of p-AKT and caspase-3 in the DT luminal site and the PT cell membrane, respectively. Scale bar = 50 μm.

The immunofluorescence staining of kidney tissues displays basal p-AKT and caspase-3 proteins expression in the control, Fund, and Alt-treated groups. The Cis group showed a marked increased expression of p-AKT and caspase-3 in the DT luminal site (blue arrows) and the PT cell membrane (yellow arrows), respectively (Figure 5) compared to the control. The Fund + Cis and Cis + Alt groups show similar expression patterns for p-AKT and caspase-3 compared to cis mice (Figure 5A,B). Fluorescence is quantified and blotted for p-AKT (Figure 5C) and caspase-3 (Figure 5D) using Image-J/ IH software (ImageJ bundled with 64-bit Java 8, 1.53t 24 August 2022, NIH, Bethesda, MD, USA).

The Cis (*p* < 0.05), Fund (*N.S.*), Alt (*N.S.*), Fund + Cis (*p* < 0.01) and Cis + Alt (*p* < 0.01) groups showed an increase in *p*-AKT (29.72%, 23.01%, 14.69%, 74.24% and 40.35%) and caspase-3 (20.04%, 5.52%, 4.03%, 29.80% and 46.80%), respectively, when equated to the control group. Both the Fund + Cis (*p* < 0.01) and Cis + Alt (*N.S*) groups showed an increase in p-AKT expression by 34.31% and 8.19% (*N.S.*), respectively, in comparison to the Cis group. Similarly, the Fund + Cis (*N.S*) and Cis + Alt (*p* < 0.01) groups showed an increase in caspase-3 expressions by 8.12% and 22.28%, respectively, when compared to the Cis group.

### 2.7. Nuclear Morphology after Treatment with Cis with/without Fund or Alt Using DAPI Staining

Apoptotic cells can be recognized by the condensation of the chromatin that occurs during the apoptotic process. Furthermore, the intensity of DAPI staining increases when the chromatin in nuclei is condensed, resulting in a reduction in the size of the nucleus, and, consequently, the nuclear area.

**Figure 6:**
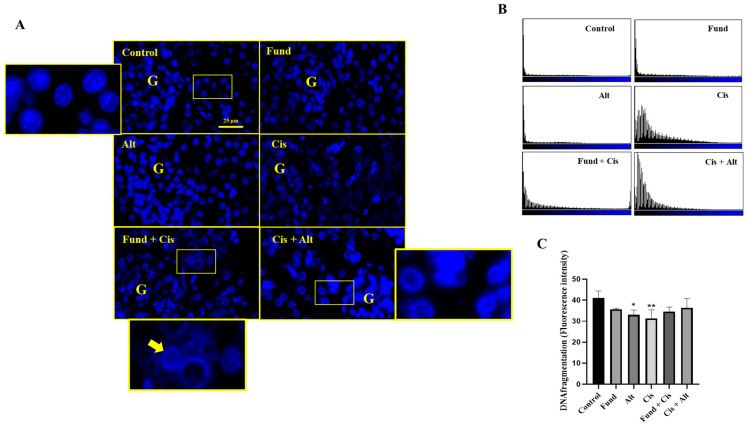
Nuclear morphology after treatment with Cis with/without Fund or Alt using DAPI staining. The rectangular yellow box zooms the area of the image referring to chromatin condensation (**A**). In addition, histogram presentation (**B**), and graphical presentation for the quantification of the fluorescence intensity (**C**). Data are expressed as mean ± SEM, where: * *p* < 0.05 and ** *p* < 0.01 are significantly different from the control group, using one-way ANOVA followed by Tukey–Kramer multiple comparisons test value. Scale bar = 25 μm.

Immunofluorescence staining using the DAPI of kidney tissues displays a typical mesh-like structure of the chromatin in the control, Fund, and Alt-treated groups. In addition, the Cis, Fund + Cis, and Cis + Alt groups showed chromatin condensation related to the apoptotic changes using a fluorescence microscope (Figure 6A,B). The DNA fragmentation intensity decreased significantly in the Cis (*p* < 0.001) and Alt (*p* < 0.05) groups by −23.82%, and −19.49 while remaining insignificantly decreased in the Fund (*N.S.*), Fund + Cis (*N.S.*), and Cis + Alt (*N.S.*) groups by −13.07%, −15–87% and −11.27%, respectively, when equated to the control group (Figure 6C). The DNA fragmentation intensity increased insignificantly in the Fund + Cis (*N.S.*) and Cis + Alt (*N.S.*) groups by 10.43% and 16.55%, respectively, compared to the Cis group.

### 2.8. Effect of Cis with/without Fund or Alt on PAR-2 Protein Expression in Mice Renal Tissues

The immunofluorescence staining of kidney tissues showed basal PAR-2 protein expression in the control, Fund, and Alt groups in the apical part in the cortical tubules (yellow arrows) and G (red arrows).

**Figure 7:**
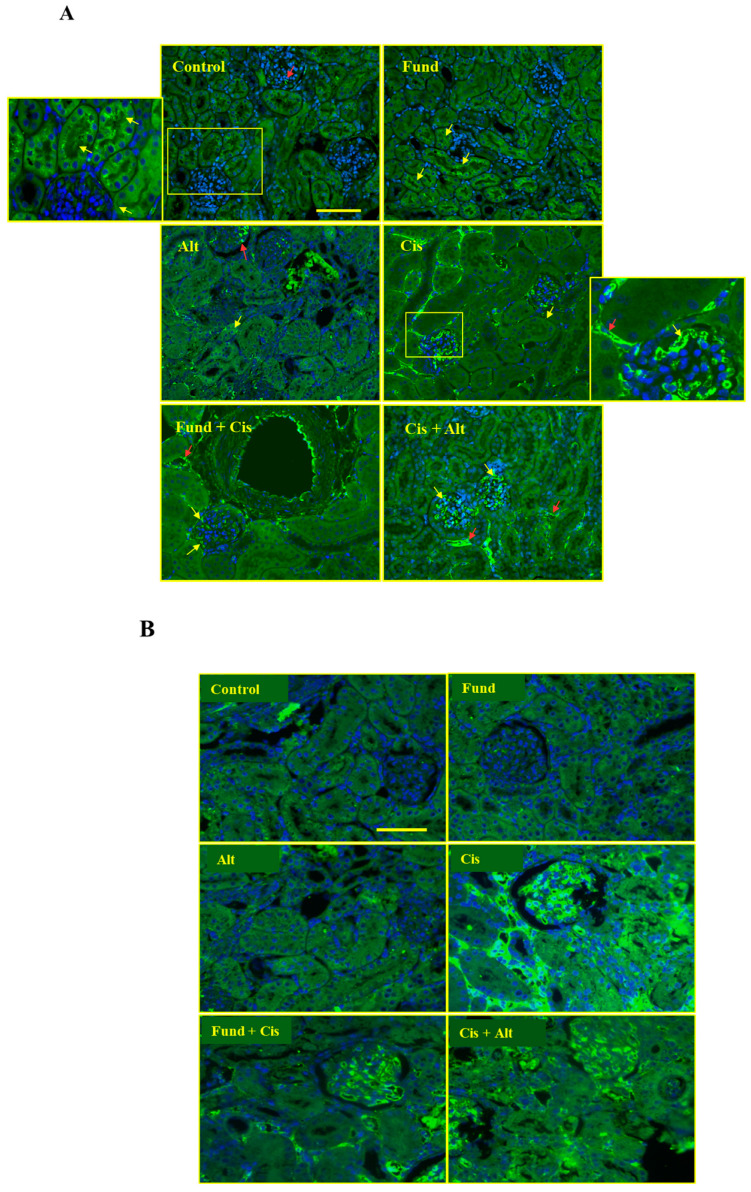
Effect of Cis with/without Fund or Alt on PAR-2 protein expression in mice renal tissues using immunofluorescence technique 200× (**A**) and 400× (**B**). A small yellow box highlights the zoomed area of the image. PAR-2 protein expression in the control, Fund, and Alt-treated groups are found in the cortical tubules (red arrow) and G (yellow arrow), while in the remaining groups, it is expressed in the migrated immune cells (red arrow) and the glomerular podocytes (yellow arrow). Scale bar = 50 μm (**A**) and 100 μm (**B**).

The Cis + Alt group shows a marked decrease in PAR-2 in the tubulointerstitial space especially in the migrated immune cells (red arrows) and in the glomerular podocytes (yellow arrows), while Fund + Cis demonstrated a reduction in PAR-2 protein expression in the tubulointerstitial space especially in the migrated immune cells (red arrows) and in the glomerular podocytes (yellow arrows) compared to the Cis group (Figure 7A,B).

### 2.9. Effect of Cis with/without Fund or Alt on Fibrinogen Protein Expression in Mice Renal Tissues

The immunofluorescence staining of kidney tissues displays basal fibrinogen protein expression in the control, Fund, and Alt groups in the tubulointerstitial space (yellow arrows) and with no fibrinogen protein expression in the G.

**Figure 8:**
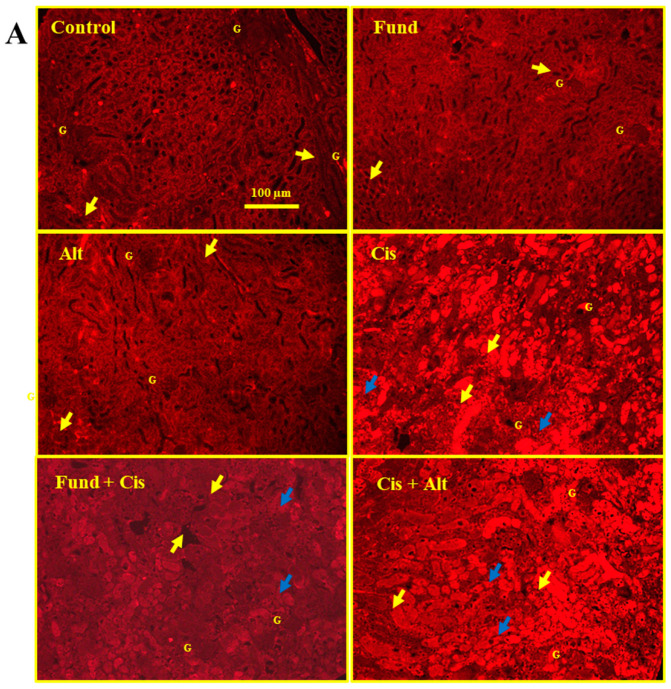

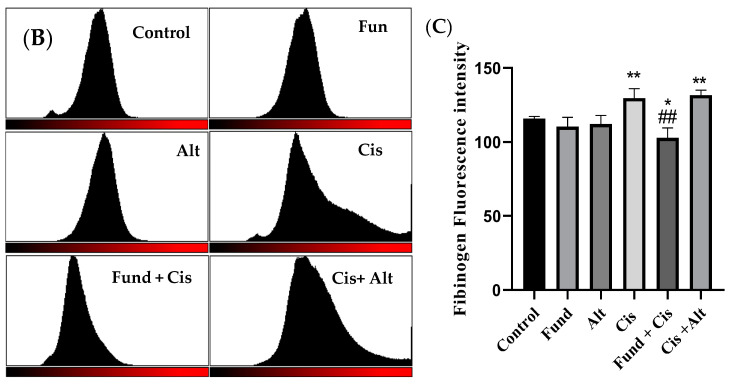
Effect of Cis with/without Fund or Alt on fibrinogen protein expression in mice renal tissues using the immunofluorescence technique (**A**), histogram presentation (**B**), and graphical presentation for the quantification of the fluorescence intensity (**C**). Data are expressed as mean ± SEM, where * *p* < 0.05 and ** *p* < 0.01 indicate significantly different from the control group, ^##^ *p* < 0.01 indicates significantly different from cisplatin mice, using one-way ANOVA followed by a Tukey–Kramer multiple comparisons test value. Fibrinogen protein expression in the control, Fund, and Alt-treated groups in the tubulointerstitial space (yellow arrow) while in the remaining groups in the DT (blue arrows) and PT (yellow arrows). Scale bar = 100 μm.

Cis- and Cis + Alt-treated mice showed a marked increase in fibrinogen in DT (blue arrows) and PT (yellow arrows). The Fund + Cis group shows a reduction in fibrinogen protein in DT and PT and the expression concentrated in the cell membrane for both DT (blue arrows) and PT (yellow arrows) (Figure 8A). A histogram shows the effect of Cis in the presence or absence of Fund or Alt on fibrinogen protein expression (Figure 8B). Fluorescence is quantified utilizing Image-J/NIH software (Figure 8C). The Cis group (*p* < 0.01) and Cis + Alt (*p* < 0.01) group showed an increase in fibrinogen expression by 11.90% and 13.63%, while the Fund (*N.S.*), Alt (*N.S.*), and Fund + Cis (*N.S*) groups decreased by −4.65%, −3.19% and −11.21%, respectively, when equated to the control group. The fibrinogen expression levels of the Fund + Cis (*p* < 0.01) and Cis + Alt (*N.S*) groups decreased and increased by −20.66% and 1.54%, respectively, when equated to the Cis group.

## 3. Discussion

The kidney plays a crucial role in the body by maintaining water–salt balance, regulating blood pressure and acid–base balance as well as excreting toxic waste products and synthesizing important hormones. This is achieved by the complex interaction of over 23 different renal cell types, including glomerular cells such as parietal epithelial cells, glomerular endothelial cells, mesangial cells, and podocytes, as well as tubular cells such as distal tubules (DT) and proximal tubules (PT), collecting ducts, macula densa, loop of Henle and connecting tubules [34]. These cells are supported by various cell membrane proteins that carry out selective functions, in response to damage, in kidney cells that can modify their phenotype to facilitate healing or shift toward the fibrotic pathway.

In the current study, Cis induced glomerular atrophy (GA), marked apoptosis in the epithelial lining PT, and partial loss of brush borders (BB). Moreover, Cis- and Cis + Alt-treated mice showed ECM deposition in the tubular interstitial spaces in the vicinity of the blood vessel and the apical part of the distal PT and DT as well as G. On the other hand, Fund reduced the histological changes and the ECM induced by Cis. The renal tubular necrosis (and frequent collapse) resulting from Cis treatment was supported by the increased ECM deposition in the apical area of DT and PT. The apical deposition of the ECM may mediate ischemia and apoptosis and finally renal tubular collapse. The effect of Fund against Cis was much stronger when compared to Alt.

In this study, it was found that the two groups, Fund and Alt, unexpectedly raised the level of uric acid. Increased serum uric acid is reported as an independent prognostic tool of favorable outcomes after acute ischemic stroke [35]. The level of uric acid which is considered a normal biological fluid contains 10-fold more antioxidants than other endogenous antioxidants [36], and it demonstrates its effect by scavenging hydrogen peroxide, peroxynitrite and hydroxyl radicals and the prevention of lipid peroxidation [37]. In addition, it binds to the resulting peroxynitrite (formed from the reaction of superoxide anion with nitric oxide) breakdown products that lead to cell injury [37]. Therefore, the increase in uric acid levels in the Fund and Alt groups correlated positively with their effect against cisplatin toxicity. On the other hand, the low glucose level in the Fund + Cis and Cis + Alt groups could be due to the apparent decrease in food intake in both groups, which in turn leads to a lower blood glucose level. Furthermore, despite the significant difference exhibited in both groups compared to Cis, the level of glucose is still in the normal range.

The A Disintegrin And Metalloprotease 10 (ADAM) family is responsible for the cleavage of many cell membrane proteins, playing a regulatory role in various processes such as angiogenesis, inflammation, and immune system activation [38,39,40]. ADAMs are also involved in the development of diseases such as Alzheimer’s, asthma, cancer, and infertility [41]. In diabetic nephropathy (DN), the expression of ADAMs is upregulated [42], while in our previous study, we demonstrated a reduced protein expression of ADAM10 in human cultured podocytes and glomeruli [16]. On the contrary, an in vitro study revealed increased podocyte CXCL16 expression due to an increase in high glucose-containing media [43]. On the other hand, another work demonstrated an increased podocyte ADAM10 expression in the G of diabetic patients [42]. Decreased ADAM10 and increased CXCL16 protein expression could be a protective response for podocytes against hyperglycemia through the scavenging of oxidated LDL by CXCL16 [43]. In our earlier work, we demonstrated increased renal expression of ADAM10 in Cis-induced ARF (30 mg/kg, i.p.) in mice in a time-dependent manner [16]. In the current work, we found an increased ADAM10 protein expression in the principal cells of the cortical PT, and its expression is increased in both principle (responsible for sodium reabsorption) and intercalated cells (regulates acid–base homeostasis) of the cortical DT injection, suggesting its pathological modulatory role in sodium reabsorption and acid–base balance during Cis toxicity. Fu et al. (2014) demonstrated that ADAM10 mediated cell apoptosis probably through the signaling of caspase-3 [44]. A protein such as ADAM10 may play a pro-apoptotic role in atherosclerotic plaques [45] and increase the phosphorylation of PI3-K and Akt. Our work found increased ADAM10 expression associated with increased caspase-3 and p-Akt in the cortical DT and PT, suggesting the PI3-K/Akt pathway’s role in ADAM10-induced apoptosis in a Cis-induced ARF.

Nuclear receptors (NRs) are a type of receptor that controls various cellular functions such as metabolism, growth, and differentiation by binding to DNA. They can be activated by small molecules such as peptides, heterocyclic compounds, cytokines, and growth factors. A total of 48 different types of human NRs act as transcription factors to regulate gene expression [18]. A nonsteroidal NR such as RXR forms a homodimer or heterodimer complex with other NRs such as VDR, RAR, PPAR, and TR. These heterodimers bind to a specific region in the DNA called the response element. There are three isoforms of RXR (α, β, and γ), and the insufficiency of the α isoform has a more negative impact on health compared to others. The silencing of RXR-α in mice causes high mortality due to heart failure, while RXR-α also plays an important role in the signaling of retinoids [46]. All RXR isoforms, especially RXR-α, are mostly localized in the distal and proximal tubules with no evidence for their expression in the glomeruli. Rexinoids are selective agonists of RXR that are used in the treatment of cutaneous T-cell lymphoma. Two FDA-approved RXR agonists are bexarotene and aliretinoin. However, human treatment with rexinoids may induce hypertriglyceridemia, which is still debatable in animal models [47].

Retinoids and their receptors play an important role in various kidney diseases, in which RXR-α and RAR-α are important during mouse nephrogenesis. Severe vitamin A deficiency can result in renal ureteral abnormalities, aplasia/hypoplasia, and horseshoe kidney, while a moderate deficiency can lead to a decreased number of nephrons during rat fetal development [48]. The loss of RXR-α is assumed to play a role in renal toxicity induced by CP, but treatment with Fund or Alt has different effects on RXR-α loss. Natural retinoids have been shown to cause renal damage in rats with mesangioproliferative glomerulonephritis [49], but retinoid agonists can preserve the glomerular architecture and reduce tubulointerstitial damage due to their anti-inflammatory and antiproliferative actions [50].

Data on the relationship between ADAM10 and the retinoid receptor RXR-α are limited. Retinoids have been shown to inhibit ADAM9 and ADAM10 [51] and induce the gene expression of ADAM10 through RXR/RAR heterodimers [52]. Two potential RXR binding sites in the ADAM10 promoter region have been identified in humans, mice, and rats [53]. Our study found that in Cis-induced ARF, increased ADAM10 expression in cortical DT and PT was associated with the loss of RXR-α and increased ADAM10-mediated apoptosis in the form of caspase-3. The kidney was partially protected by Fund through reducing ADAM10, caspase-3, and p-Akt protein expression and preventing the loss of RXR-α. However, Alt increased ADAM10, caspase-3, and p-Akt protein expression and further loss of RXR-α in cortical DT and PT, suggesting that the protective effect of Fund is more significant than the treatment effect of Alt against Cis-induced ARF.

The blood coagulation system plays an important role in controlling blood hemostasis and is involved in various diseases [54]. Coagulation proteases can control renal cell function by activating surface receptors such as PARs [55,56]. In the present study, Cis displayed an increased PAR-2 in the cortical tubules including DT and PT. In addition, a constitutive fibrinogen protein expression (the end product of the coagulation system activation) was found in the G, DT, and PT. PARs are part of the seven transmembrane domain G protein-coupled receptor with four mammalian members of the PAR family PAR1-4. Although mouse and human PAR-2 genes are symmetrical to PAR1 genes, PAR-2 is not thrombin responsive. Several works have revealed that tissue factor (TF) VIIa activates PAR-2 [57,58] by the irreversible shedding of PAR-2 [59]. Moreover, PAR-2 has an important role in the interaction between coagulation and inflammation [60], resulting in the release of pro-inflammatory mediators and TF expression that leads to irreversible fibrinogen formation [61].

According to the best of our knowledge, no data exist about the relation between PAR-2, fibrinogen, and ADAM10 in general and in Cis-induced ARF in particular. We aimed in our work to find out the possible relation between PAR-2, fibrinogen and ADAM10 in Cis-induced ARF and the possible protective effect of the anticoagulant, fund, or the possible treatment of the fibrinolytic agent, Alt, in Cis-induced renal failure. Previous reports demonstrated PAR-2′s upregulation after using inflammatory mediators such as lipopolysaccharide (LPS), TNF-, IL-1, and PAR-2 agonists [62,63]. During inflammation, PAR-2 controls responses and has a role in directing generated proteolytic enzymes in infectious states [64]. Another study demonstrated that PAR-2′s activation led to increased ADAM10 protein expression in VK2 cells [65]. Furthermore, αIIbβ3-independent washed platelet aggregate formation and ADAM10 activity are increased by fibrin [66]. In conclusion, in the current study, activation of the coagulation cascade (in the form of PAR-2 and fibrinogen) by Cis treatment may induce ADAM10 in the PT and DT, leading to the cleavage and therefore loss of RXR-α and activation of apoptosis (represented by caspase-3) through the PI3-K/Akt pathway. Furthermore, Fund showed a better effect against Cis-induced ARF.

## 4. Materials and Methods

### 4.1. Drugs and Chemicals

All chemicals were obtained from Sigma-Aldrich (St. Louis, MO, USA) unless specifically specified. Cisplatin (Cis, 50 mg/100 mL) [67] and alteplase (Alt, 50 mg powder) [68] were kindly obtained from Prince Sultan Military Medical City (Riyadh, Saudi Arabia), while fondaparinux (Fund) [69] was purchased from GlaxoSmithKline Pharmaceuticals (Brentford, UK).

### 4.2. Animals

Sixty male Balb/c mice (22 ± 2 weeks old, weighing 27–30 g) were obtained from the Animal Facility, College of Pharmacy, Qassim University. The animals were housed in individual polyacrylic cages (3 mice/cage) with a chow diet (First Milling Company in Qassim, Buraydah, Saudi Arabia), and water ad libitum, 7 days before the start of the experiments. The animals were maintained at RT (~25 °C) and relative humidity of ~65% with a controlled light–dark cycle of 12:12 h. The study design was approved by the Ethics Committee of Deanship of scientific research, Qassim University, Qassim, Saudi Arabia (23-39-09), and experiments were performed by the NIH Guidelines for the Care and Use of Lab Animals.

### 4.3. Experimental Design

Mice were randomly divided into six groups (n = 10 mice/group) [70]. The first 4 groups involved control, Cis-induced ARF (Cis), Alt, and Cis + Alt groups receiving normal saline for 10 days. All 4 groups except the control received Cis (30 mg/kg, i.p) [67] on day 7 [68], and 6 h later, both the Alt groups received Alt (0.9 mg/kg, i.v) [71]. The last 2 groups (Fund and Fund + Cis) received Fund (5 mg/kg, i.p) [72] for 10 days, and the Fund + Cis group on day 7 received Cis (30 mg/kg, i.p). All the animal groups were euthanized on the 10th day or 72 h after the dose of Cis. A separate experiment with similar animal groups was conducted for measuring BW, kidney–BW ratio and prothrombin time (PT).

### 4.4. Blood Sampling and Preparation

Under anesthesia, using Thiopental (40 mg/kg), 1 mL of whole blood was collected from the retro-orbital plexus in an EDTA tube. Then, 20 µL was aspirated for complete blood count (CBC), the remainder was centrifuged (1792× *g* for 15 min) and the supernatant was obtained to analyze creatinine, urea and uric acid (Diamond Laboratory Reagents, Holliston, MA, USA) using the spectrophotometric method (JENWAY, 6305 Model, Stone, UK). In a separate experiment, the BW and kidney–BW ratio were determined in addition to PT (LABiTec GmbH, Cat # 210-08-010-00, Ahrens Burg, Germany) in citrated blood (0.9 whole blood + 100 µL citrate) using a semi-automated thrombostat coagulometer (22851 Norderstedt Germany). The animals were euthanized using cervical dislocation.

### 4.5. Tissue Sampling and Calculation of Relative Kidney Weight

Both kidneys were collected and washed with sterile ice-cooled saline (0.9%). Each dried kidney was weighed for calculation of the relative kidney weight.

**Relative potency** = Weight of kidney (gm)/Body weight of mice × 100

Tissues were then dissected, fixed immediately in Davidson’s solution, and embedded in paraffin blocks for immunofluorescence and histological analysis detection of RXR-α, ADAM10, caspase-3, fibrinogen, p-Akt, and PAR-2. Briefly, tissues were kept in Davidson’s for 24 h, which was followed by 70% alcohol. Samples were dehydrated through gradient alcohol concentrations of 50%, 70%, 95%, and 100%, followed by immersion in xylene and then paraffin embedding at 56 °C.

### 4.6. Determination of Creatinine Level

Creatinine levels in samples were determined according to the method described [73].

### 4.7. Determination of Complete Blood Count (CBC)

Hematological parameters were evaluated using a VABIO360 Auto Hematology Analyzer (BIOTA, Istanbul, Turkey). Briefly, the collected blood was gently mixed, aspirated with a needle, and counted using the automated instrument.

### 4.8. Histopathological Analysis

Serially sectioned paraffin-embedded kidney sections (4 µm size) using microtome were obtained on glass sides and deparaffinized followed by hematoxylin and eosin stain. The sections were analyzed under a light microscope [74] by a pathologist blinded to the experiment. The grade of renal injury was ranked into five grades (0 = no injury; 1 = minimal (<1%); 2 = slight (1–25%); 3 = moderate (26–50%); 4 = moderate/severe (51–75%); 5 = severe/high (76–100%).) and was rated from 0 to 5 [75]. Three sections/mice were analyzed for histopathology, and one researcher blinded for experimental groups carried out the analysis to avoid any bias.

### 4.9. Single Immunofluorescence Analysis of ADAM10, Fibrinogen, RXR-α and PAR-2 in Renal Tissue Sections

Tissue sections were processed according to the following steps [76]. Briefly, slides were kept in the oven (60 °C) for 20 min followed by deparaffinization using xylene (100%) and gradual hydration with absolute ethanol, ethanol (90%), (75%), (50%), (30%), and distilled water for 5 min/each. The antigen retrieval step was conducted with Dako^®^ citrate buffer (pH 6.0) in the microwave (500 watts for 20 min) followed by washing with 0.05% tween 20 in phosphate-buffered saline and tissue fixation using methanol (10 min). Slides were blocked using blocking solution (1% BSA, 10% horse serum in 1× in PBS) for 1 h at room temperature (RT) followed by incubation (3 h at 37 °C and overnight at 4 °C) with the appropriate primary antibodies (mouse anti-ADAM10 with cat # sc-28358, Fibrinogen with cat # sc-65966, RXR-α with cat # sc-515929 and PAR-2 with cat # sc-13504, diluted 1:150 in blocking solution, Santa Cruz Biotechnology, Dallas, TX, USA). After washing, the slides were incubated (37 °C for 30 min) with cyanine red (Cy3) conjugated goat anti-mouse (Invitrogen, cat # M30010, Carlsbad, CA, USA) for fibrinogen, RXR-α and PAR-2 or Alexa 488 conjugated goat anti-mouse (Invitrogen, cat # A31620, Carlsbad, CA, USA) for ADAM10 secondary antibodies. The slides were rewashed with 0.05% tween 20/1× PBS 2 times, counterstained for 3 min with 4′,6-diamidino-2-phenylindole (DAPI, Sigma Aldrich, cat # D954, St. Louis, MO, USA), and mounted using fluoromount^®^ mounting solution (Sigma Aldrich, cat # F4680, St. Louis, MO, USA). Tissue sections were analyzed, and the images were captured using a Leica fluorescence microscope (Model: Leica DM 5500B, Leica Microsystems, Wetzlar, Germany). A minimum of 6 fields per mouse was used in the fluorometric analysis using Image-J software NIH.

### 4.10. Double Immunofluorescence Analysis of Caspase-3 and p-Akt in Renal Tissue Sections

Tissue sections were prepared as described above followed by incubation (3 h at 37 °C and ON at 4 °C) with mouse anti-p-Akt (cat # 4051) and rabbit polyclonal anti-caspase-3 primary antibodies, 1:150 in blocking solution (Cell Signaling Technology, cat # 9662, MA, USA). After washing, the slides were incubated (37 °C for 30 min) with Cy3 conjugated goat anti-mouse (for p-Akt) and Alexa 488 conjugated goat anti-rabbit (Invitrogen, cat # A-11008, Carlsbad, CA, USA) secondary antibodies for caspase-3. The procedure was then followed as described above.

### 4.11. Trichrome Stain

Following the manufacturer’s instructions, tissues were stained for ECM detection using a trichrome stain (Masson) kit (Sigma Aldrich, cat # HT-15, St. Louis, MO, USA). In brief, deparaffinized tissue slides were incubated in preheated Bouin’s solution at RT. Slides were washed in running tap water and stained in Weigert’s Iron Hematoxylin Solution (5 min). Washed tissue sections were stained in Biebrich Scarlet-Acid Fuchsin (5 min) and rinsed in deionized water. Sections were placed in a phosphotungstic/phosphomolybdic acid solution (5 min) and were placed in an aniline blue solution (5 min). Slides were placed in acetic acid, 1%, for 2 min followed by rinsing in tap water; then, the slides were dehydrated through alcohol and cleared in xylene and finally mounted for further analysis under a microscope.

### 4.12. Statistical Analysis

Data are expressed as mean ± SEM, *p* < 0.05 was considered statistically significant. One-way ANOVA followed by a Tukey–Kramer multiple comparisons test was used to analyze differences between groups using GraphPad Prism (8.0.2), USA. Data passed homogeneity/normality using Anderson–Darling, D’Agostino and Pearson, Shapiro–Wilk and Kolmogorov–Smirnov tests.

## 5. Conclusions

Our work, for the first time, reported that ADAM10, PAR-2, caspase-3, p-Akt, and fibrinogen protein expression are increased and RXR-α expression is decreased in Cis-induced ARF in mice. The current results suggest that activation of the coagulation system takes place through the activation of PAR-2 and fibrin as a result of Cis-induced ADAM10 protein expression that mediates the apoptotic pathway as indicated by caspase-3 through p-Akt. This is normally accompanied by the loss of RXR-α distal and proximal tubules as lipid droplets. Pre-treating mice with the anticoagulant, Fund, halted the previous deleterious effect, while the fibrinolytic agent, Alt, most of the time has failed to treat the consequences of Cis injection.

## Figures and Tables

**Table 1:** Effect of Fund or Alt on WBCs, RBCs, platelets count, prothrombin time and glucose in Cis-induced ARF.

	Control	Fund	Alt	Cis	Fund + Cis	Cis + Alt
**WBCs (10^9^/L)**	5.613 ± 0.12	5.047 ± 0.04 ***	5.147 ± 0.35 ***	4.253 ± 0.14 **	2.973 ± 0.21 ***^, ###^	1.727 ± 0.13 ***^, ###^
**RBCs (10^6^ cell/L)**	5.467 ± 0.05	5.353 ± 0.08	5.893 ± 0.06	6.283 ± 0.09 ***	5.433 ± 0.11 ^###^	5.5 ± 0.1 ^###^
**PLT (10^9^/L)**	795 ± 14.47	737 ± 9.29 **	288 ± 10.6 ***	463.3 ± 13.54 ***	730 ± 8.88 **^, ###^	334.3 ± 5.78 ***^, ###^
**Prothrombin time (s)**	8.6 ± 0.31	10.96 ± 0.26 ***	10.60 ± 0.24 ***	14.98 ± 0.09 ***	11.54 ± 0.31 ***^, ###^	11.38 ± 0.23 ***^, ###^
**Glucose (mg/dL)**	115 ± 9.640	117.7 ± 10.97	113.5 ± 9.926	142.9 ± 13.54	77 ± 10.38 ^###^	62.33 ± 3.930 *^,###^

Data are represented as means ± SEM (n = 10). WBCs, white blood cells; RBCs, red blood cells; PLT, platelets. Here, * *p* ≤ 0.05, ** *p* < 0.01, and *** *p* < 0.001 were statistically significant when equated to the control group, while ^###^ *p* < 0.001 were statistically significant when equated to the cisplatin-treated group using one-way ANOVA followed by a Tukey–Kramer post-test for multiple comparisons (*p* < 0.05).

## Data Availability

The data used to support the findings of this study are included within the article.
